# Diagnostic errors by medical students: results of a prospective qualitative study

**DOI:** 10.1186/s12909-017-1044-7

**Published:** 2017-11-09

**Authors:** Leah T. Braun, Laura Zwaan, Jan Kiesewetter, Martin R. Fischer, Ralf Schmidmaier

**Affiliations:** 1 Institut für Didaktik und Ausbildungsforschung in der Medizin, Klinikum der LMU München, Ziemssenstr. 1, 80336 München, Munich, Germany; 2 Medizinische Klinik und Poliklinik IV, Klinikum der Universität München (LMU), Munich, Germany; 3 Institute of Medical Education Research Rotterdam (iMERR), Erasmus MC, Wytemaweg 80, 3015 CN Rotterdam, the Netherlands

**Keywords:** Diagnostic errors, Undergraduate medical education, Clinical reasoning

## Abstract

**Background:**

Diagnostic errors occur frequently in daily clinical practice and put patients’ safety at risk. There is an urgent need to improve education on clinical reasoning to reduce diagnostic errors. However, little is known about diagnostic errors of medical students. In this study, the nature of the causes of diagnostic errors made by medical students was analyzed.

**Methods:**

In June 2016, 88 medical students worked on eight cases with the chief complaint dyspnea in a laboratory setting using an electronic learning platform, in summary 704 processed cases. The diagnostic steps of the students were tracked and analyzed. Furthermore, after each case the participants stated their presumed diagnosis and explained why they came to their diagnostic conclusion. The content of these explanations was analyzed qualitatively.

**Results:**

Based on the diagnostic data gathering process and the students’ explanations, eight different causes could be identified of which the lack of diagnostic skills (24%) and inadequate knowledge base (16%) were the most common. Other causes that often contributed to a diagnostic error were faulty context generation (15%) and premature closure (10%). The causes of misdiagnosis varied per case.

**Conclusions:**

Inadequate skills/knowledge and faulty context generation are the major problems in students’ clinical reasoning process. These findings are valuable for improving medical education and thus reducing the frequency of diagnostic errors in students’ later everyday clinical practice.

## Background

Errors in medicine are frequent [1, 2] and put patients’ safety at risk [3]. For example, the frequency of major diagnostic errors uncovered during autopsies [4] is about 8.0–22.8%. The causes of diagnostic errors in Internal Medicine have been classified by Graber et al. in an analysis of 100 diagnostic error cases [5]. Specifically, diagnostic errors were identified through autopsy discrepancies, quality assurance activities, and voluntary reports. By analyzing those errors, a working taxonomy for cause-classification was developed: In this taxonomy system errors and cognitive errors and no-fault errors are distinguished [5]. Whereas system-related errors or situational errors are problems in communication structure or procedures, the cognitive errors belong to the field of clinical reasoning. Cognitive factors account for three quarters of all diagnostic errors in Internal Medicine, either solely or in combination with system-related factors [5]. Cognitive errors can be further subdivided into a) faulty knowledge, b) faulty data gathering, and c) faulty synthesis (faulty information processing and faulty verification). Each of these categories can be subdivided further to describe precisely the individual aspects of the cognitive process error [5]. Concentrating on the cognitive errors, premature closure is the most common reason for mistakes by physicians, as has been shown in different studies [5, 6]. One explanation for premature closure could be found in the dual processing theory [7, 8]. The dual processing theory is widely accepted as an explanation for cognitive processes in clinical reasoning. It describes that cognitive processes are governed by so called system I (which is intuitive, automatic, fast, narrative, experiential and affect-based) and system II (which is analytical, slow, verbal, deliberative and logical) [9, 10]. However, the explanation that premature closure is a problem associated with the application of system-I thinking might oversimplify the problem [11]. These findings have been critically discussed as the relationship between knowledge and premature closure may be unclear [12].

Most studies in which diagnostic errors are analyzed focus on physicians. Studying students’ diagnostic errors is important as well. First, it can provide understanding of the etiology of diagnostic errors. Second, it will provide insights for improving medical education on clinical reasoning during medical school. Nevertheless, only little is known about cognitive clinical reasoning errors made by medical students. Using standardized patients, Friedman et al. found that students often used non-discriminating findings to support a diagnosis [13]. Elstein et al. described a similar error type: the over-interpretation/ under-interpretation and misinterpretation of findings [14]. Premature closure is also an issue in medical students’ clinical reasoning and appears frequently [6]. We have previously shown that additional knowledge beyond a solid factual knowledge base is not correlated with increased diagnostic competence [15]. This is in line with results that revealed that the application of different kinds of knowledge is not correlated with the diagnostic performance [16].

In summary, while some studies exist, there is a need for a more comprehensive investigation into diagnostic errors of medical students. This would enable the development of novel teaching strategies which can improve the clinical reasoning process of medical students, the future medical practitioners. We examined the cognitive causes of diagnostic errors in medical students using the categories of Graber et al. [5] and determined to what extent the frequency of the types of causes differ from those of experts. Therefore, the main research question is: What are the causes of diagnostic errors of medical students?

## Methods

### Design and participants

We present a computer-based laboratory study to qualitatively describe the diagnostic errors of medical students. The study is the qualitative part of a larger research project dealing with diagnostic competence. The quantitative part concerning the effectiveness of scaffolding to foster diagnostic efficiency is reported elsewhere [17]. 

In June 2016, 88 4th and 5th year medical students from two medical schools in Munich (Ludwig-Maximilians-University and Technical University) participated in this study. Years 1 and 2 of medical school are identical for all Munich medical students. Years 3 to 5 of the both medical schools may differ in teaching strategies, but not in content as both medical faculties have to teach under the national legislation that defines all clinical clerkships and regulates the hours of clinical teaching. Of all participants, 20% were students from the Technical University. They received a financial incentive for participating. The Ethical Committee of the Medical Faculty of LMU Munich approved the study.

### Study environment and procedure

**Fig. 1:**
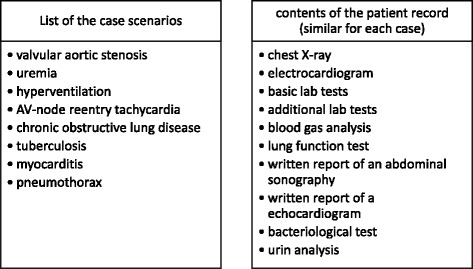
List of the cases and contents of the electronic patient record

First, the students completed a socio-demographic questionnaire and a test on content-specific pre-knowledge. Then they worked within the electronic case simulation platform CASUS [18] on eight clinical cases in Internal Medicine (diagnoses see Fig. 1) with dyspnea as chief complaint. Each case consisted of a medical history, a physical examination and an electronic patient record (contents see Fig. 1). Participants could freely select the number and sequence of information from the electronic learning platform that they regarded as important to diagnose the case. The information from the history and the physical examination and the number of selected technical examinations was not restricted and the students could choose as many examinations as they wanted. However, this was restricted to the amount of information that was available in the electronic learning platform. The amount of available information was quite extensive: The history provided the following information in each case: sex, age, pre-existing conditions, medication, alcohol- and nicotine-abuse, history of present illness, symptoms. The physical examination included information regarding the vital signs, the general and nutrition condition, an examination of the cardiovascular-system, the abdomen, the lung, the lymph nodes and a neurological examination. The 10 technical examinations are listed in Fig. 1. Also, the sequence in which the students assessed the history, the physical examination and the technical examinations was completely up to them. Additionally, they were allowed to go back to any of this information as often as they wanted. In the end, they were required to state their final diagnosis. No feedback was given on their diagnoses. After each case, participants had to write an explanation why they had chosen their diagnosis. Importantly, in the case scenarios developed for this study the diagnostic knowledge and clinical reasoning abilities as well as the diagnostic skills of the participants (such as interpretation of electrocardiograms, lung function tests and x-rays) were examined, and thus allowing for a more specific assessment of diagnostic errors.

The cases were written by the study author (LB) based on material from real cases. Five resident physicians and attending doctors reviewed the cases. In a pilot study, 10 students evaluated the feasibility and comprehensibility of the study procedure and the difficulty of the cases. The students participating in the pilot study did not take part in the actual study.

### Analysis of the accuracy and the diagnostic steps

Diagnoses were binary rated as correct or incorrect according to an expert solution of the case.

The learning environment CASUS is organized in screens with different contents. The history (in one screen) the physical examination (in one screen), and each of the 10 technical examinations was put in a separate screen, respectively. Altogether, there were 12 different screens with clinical information. CASUS tracks which screen is selected by the participant, when and for how long. The participants could freely navigate through the system and select screens of interest. Thus, the sequence of the information gathering process of each participant was individually tracked in detail. Therefore, we were able to detect if a participant had missed an important piece of information that was needed to solve the case correctly.

### Content analysis

We used the content analysis according to Mayring [19] and thus investigated the explanations qualitatively. The explanations were evaluated to determine the main cognitive causes of medical students’ diagnostic errors. These causes were categorized. Finally, each explanation was assigned to a category. One investigator (LB) coded all explanations. A second rater coded 11% of the explanations. The interrater coefficient analyzed with Cohens kappa was k = 0.859.

## Results

88 (58 female) participants processed all cases and their diagnoses were analysed. The mean age was 24.6 years (SD = 0.48) and they had on average spent 14.9 weeks (SD = 0.48) on clerkships and block placements. There were not significant differences between the participants form the two Munich medical faculties regarding any of the following results.

### Results of the content analysis: Frequency, distribution and nature of errors

Participants misdiagnosed 304 out of 704 times.

The errors could be divided into two main categories: faulty knowledge and faulty information processing. Table 1 shows the error types with a slightly modified definition of Graber [5] and an error example.

**Table 1:** Error types and examples

Type	Definition [5]	Example
Knowledge base inadequate	Insufficient knowledge of relevant condition	*“Poor general and nutritional condition with fever. Positive (blood) culture with acid-resistant rods. Unfortunately, I can’t remember which pathogen this kind of staining indicates.”* (diagnosed: infection, correct diagnosis: tuberculosis)
Skills inadequate	Insufficient diagnostic skills for relevant condition	*“….no hint for pneumothorax or pneumonia”* (diagnosed: viral infection, correct diagnosis: pneumothorax)
Faulty context generation	Lack of awareness of relevant aspects of the case	*“patient has dyspnea and inflammatory markers”* (diagnosed: viral infection, correct diagnosis: COPD)
Overestimating/ underestimating	Focus too closely on an aspect or failure to appreciate the relevance	*“He also has a renal insufficiency – this might explain the nausea. But the atrial fibrillation explains the dyspnea”* (diagnosed: atrial fibrillation, correct diagnosis: uremia)
Faulty triggering	Inappropriate conclusion	*“Infection of the upper airways, pericardial effusion”* (diagnosed: viral airway infection, correct diagnosis: myocarditis)
Misidentification	One diagnosis is mistaken for another	*“… post-streptococcal endocarditis. History of infection and ST-segment elevation in the electrocardiogram”* (diagnosed: endocarditis, correct diagnosis: myocarditis)
Premature closure	Failure to consider other possible diagnosis	*“the risk factors, the acute onset of symptoms and the young age of the patient are indicative for a pulmonary embolism”* (diagnosed: pulmonary embolism, correct diagnosis: hyperventilation)
Cluelessness	Failure to find any diagnosis at all	*“based on the given information I could not find a diagnosis, it could be an iron deficiency anemia but this would not explain the acute onset (….)”* (diagnosis missing, correct diagnosis: AV-node-reentry-tachycardia)

Altogether, inadequate skills led to the most diagnostic errors. When considering the information processing, faulty context generation is the most common cause of error (Table 2).

**Table 2:** Diagnostic errors of medical students

Type	Frequency (in %)
Knowledge base inadequate	16% (49/304)
Skills inadequate	24% (75/304)
Faulty context generation	15% (47/304)
Overestimating/underestimating	9% (28/304)
Faulty triggering	12% (35/304)
Misidentification	10% (30/304)
Premature closure	10% (29/304)
Cluelessness	3% (9/304)

The distribution of the categories differs greatly between the cases: In the cases tuberculosis, COPD, pneumothorax and AV-node-reentry-tachycardia (AVNRT), faulty knowledge (knowledge and skills) is the most common error, whereas faulty information processing and misidentification are more relevant in the cases myocarditis, uremia and hyperventilation (Table 3).

**Table 3:** Most common diagnostic errors with respect to the different cases

Case	Total number of errors (of 88)	Most common error	Percent
Tuberculosis	51	Faulty knowledge	45% (23/51)
Pneumothorax	45	Faulty skills	42% (19/45)
Myocarditis	28	Faulty context generation, Misidentification	36% each (10/28)
Valvular aortic stenosis	3	Faulty context generation	66% (2/3)
COPD	32	Faulty skills	47% (15/32)
Uremia	33	Faulty context generation, Overestimating	30% each (10/33)
Hyperventilation	43	Misidentification	30% (13/43)
AVNRT	72	Faulty skills	38% (27/72)

We were able to identify seven of Graber’s 25 cognitive categories [5] in our material but added the category “cluelessness” to categorize missed diagnoses. The category “Faulty detection or perception” was combined with the category “faulty skills”. Also, the category “Confirmation bias” and failed heuristics were considered to be a part of premature closure.

### Results of the diagnostic steps analysis

In addition to analyzing the content, we used the information from the analysis of the diagnostic steps to classify the error categories more precisely. By tracking the diagnostic steps, we could analyze if the students had missed diagnostic information that would have been needed to solve the case correctly. For example, the case myocarditis could only be solved correctly by looking at the echocardiogram. Since we were able to obtain insights in both the diagnostic information that was selected by the students as well as their reasoning process, we were able to identify the root causes of the diagnostic errors. Specifically, in Graber’s system [5], faulty data gathering is a separate error category. In our study, we were able to further analyze those errors and determine whether omitting to gather an important piece of diagnostic information was due to faulty knowledge (the student does not know which diagnostic information is needed), faulty context generation (the student is not aware of the importance of the examination in this specific situation) or premature closure (the student is not considering a diagnosis for which this examination is relevant). As the material shows, faulty data gathering occurred in all cases (Table 4). Interestingly, it especially occurred in cases in which a slightly unfamiliar technical examination was needed to solve the case. The students tended to almost always look at the chest x-ray, the lab test and the electrocardiogram but frequently skipped the lung function test (needed to solve the case COPD), the bacteriological test (important for solving the case tuberculosis) or the echocardiogram (for the case myocarditis).

**Table 4:** Faulty data gathering (referred to the misdiagnoses)

Case	Total number of misdiagnoses in that case	Proportion of students who missed an important piece of diagnostic information (%)
Tuberculosis	51	27 (53%)
Pneumothorax	45	6 (13%)
Myocarditis	28	19 (68%)
Valvular aortic stenosis	3	3 (100%)
COPD	32	18 (56%)
Uremia	33	4 (12%)
Hyperventilation	43	15 (35%)
AVNRT	72	10 (14%)

## Discussion

### Importance and goal of this study

The problem of diagnostic errors is undeniable and studies are needed to understand the causes for diagnostic errors which can inform educational programs and interventions to reduce diagnostic errors. The aim of this study was to describe the nature of the causes of diagnostic errors by medical students and determine if error categories used to analyze experts’ diagnostic errors can be transferred to a population of medical students.

### Summary and discussion of our results

We found eight different error categories representing the root causes of the diagnostic errors. When those categories are compared to the causes that Graber [5] found in experts, large differences can be detected: students misdiagnose far more often due to faulty knowledge and/or skills. The amount of information about the reasoning process that was available in our study was likely more detailed than in the study of Graber et al. [5]. It could be that in the study of Graber et al. [5] actual knowledge deficits could not be revealed with the information at hand and were therefore attributed to other causes such as failed heuristics or premature closure. On the other hand, it is not surprising that students have less knowledge than experts. However, the students had recently finished the Internal Medicine curriculum and were therefore expected to have a sound knowledge base of the relevant facts. For instance, they should have learned which staining method is used to identify common pathogens during their Internal Medicine curriculum but often they did not know that a positive Ziehl-Neelsen staining indicates tuberculosis. This raises concerns about the sustainability of the factual knowledge that was passed on to the students in their Internal Medicine curriculum [20]. Many studies about fostering diagnostic accuracy focus on improving reflection methods, although the first and fundamental steps are knowledge and skills. Interestingly, some studies indicate that more knowledge does not lead automatically to a better case solution [15, 16]. Nevertheless, based on our study knowledge-based solutions to decrease diagnostic errors seem appropriate.

Knowledge gaps should be identified, and learning how to apply diagnostic knowledge and skills should be systematically learned with the help of virtual clinical cases and throughout clinical clerkships.

A lack of diagnostic skills such as the correct interpretation of an electrocardiogram or an x-ray was identified as a major cause of diagnostic errors. The results do not differ between the two medical schools although the curricula substantially differ (data not shown). Not only medical students but junior doctors as well show poor competence in the interpretation of x-rays [21, 22]. We were able to replicate these findings; evidently, these skills - or at least the application of these skills in clinical cases - are not sufficiently trained in medical school. Students need to learn these skills through a lot of repetitive practice. Furthermore, there might be a gap between the clinical skills as taught and daily clinical practice. At some point, students should be confronted with more complex and atypical results of technical examinations and not only with classic textbook cases. A more structured approach or checklists might help to improve the diagnostic skills [23].

Premature closure is also an issue among medical students although it is not the most common cognitive error in this group. This kind of error seems to get more and more dominant with growing expertise as other error categories become less important [5]. Specific information within the medical history seems to result in premature closure. For example, many students concluded a lung embolism from the information “intake of an oral contraceptive”. Similarly, the information “nicotine abuse” misled students to diagnose a lung cancer although this diagnosis was not supported by the technical examinations. Possibly students are trained to recognize salient clues that they do not longer consider other possibilities. Norman et al. describe premature closure as a potential result of insufficient knowledge: insufficient knowledge reduces the availability of other diagnoses, and thus premature closure seems to be the error [12], whereas in fact it is not. Viewed from this angle, our results might shed some light on the development of cognitive errors in clinical reasoning: First, students have faulty or insufficient knowledge, then their faulty or insufficient knowledge might lead to concealed premature closure. In the case of experts, however, premature closure could be a result of overconfidence in their own diagnosis [24]; a lack of knowledge seems only rarely to be the reason for diagnostic failure [5]. In contrast, other studies have underlined that insufficient knowledge is the main cause of diagnostic errors in experts, and premature closure is caused by insufficient knowledge [25, 26].

To reveal the connection between insufficient knowledge and premature closure, a prospective study is needed to investigate long-term developments. If premature closure is a result of faulty knowledge, it should decrease with growing knowledge. However, premature closure increases with expertise level. Premature closure is still a black box. Using think-aloud-protocols in further studies might help to answer these questions.

As shown, the reasons for diagnostic errors are highly dependent on the specific case. Whereas some cases provoke errors due to faulty skills, others might lead to premature closure. This might be due to the fact that the crucial cognitive reasoning step is case specific: for instance, in COPD or AV-node-reentry-tachycardia (AVNRT) cases a technical examination needs to be interpreted correctly (lung function test and ECG respectively). Inadequate skills hamper correct case solution. On the other hand, in syndrome cases like hyperventilation many (positive and negative) results need to be weighed, which stresses competences like information processing and identification. This characterization of cases might help to foster diagnostic competence in a more specific way: As is already known, learning with cases and learning from mistakes [27] is helpful. Studies are needed to investigate whether the avoidance of a specific error could be taught by utilizing cases typically inducing specific errors and then reprocessing the error path.

### Strengths and limitations

Our study has several strengths. First, the diagnostic process remained uninterrupted and the diagnostic outcome was fully available to our investigations. Second, we recorded the clinical reasoning process and the diagnostic errors in a controlled and prospective setting. Contrary to other studies, we could analyze the reasoning process and track the diagnostic process and therefore we were able to obtain more insights into the underlying causes of diagnostic error. A relevant problem in the investigation of diagnostic errors is the so-called hindsight bias [28, 29]. Knowing that a diagnosis is wrong, increases the probability to find errors in the diagnostic process. In our laboratory setting, the correct diagnostic process and explanations were established before starting to collect the data. In this way, we tried to avoid hindsight bias. Additionally, we were able to identify the root causes of the diagnostic errors. We were able to do this, contrary to other studies, as this study was prospective using a laboratory setting. We obtained information about the reasons for the participants’ diagnostic conclusions as well as whether they had looked at certain pieces of diagnostic information. Especially for improving medical education, it is important to understand the root causes of diagnostic error because that way, it will be easier to find fitting instructional interventions to reduce the frequency of errors made by medical students. We suggest fostering the diagnostic skills of medical students by using well-designed cases with explicit feedback on the technical interventions. Students need to practice the transfer of their diagnostic knowledge in clinical-case settings. Further, they should also be, depending on their educational-level, confronted with atypical and more uncommon findings in technical examinations.

A limitation of the study is that we, like in the study of Graber et al. [5], only used Internal Medicine cases. Further studies are needed to analyze whether the categories can be transferred to other medical disciplines. Also, our study included 8 different cases and it remains unclear whether the same error categories can be found in a sample of more cases. Though, we investigated a large sample size, thus gaining greater range of answers, all students were recruited from only two medical school. This might have led to a bias with respect to the curriculum related knowledge base.

## Conclusions

This study showed that the main causes of diagnostic errors in medical students involve inadequate skills (and knowledge base) and faulty context generation. Based on these results, we propose further research on how to address these problems by instructional interventions and a longitudinal assessment to see the changes, when the knowledge base increases.

## Abbreviations

AVNRT: AV-node-reentry-tachycardia
COPD: Chronic obstructive pulmonary disease
ECG: Electrocardiogram
LB: Leah Braun
LMU: Ludwig-Maximilians-University
